# The Halophile Protein Database

**DOI:** 10.1093/database/bau114

**Published:** 2014-12-01

**Authors:** Naveen Sharma, Mohammad Samir Farooqi, Krishna Kumar Chaturvedi, Shashi Bhushan Lal, Monendra Grover, Anil Rai, Pankaj Pandey

**Affiliations:** Center for Agricultural Bioinformatics, Indian Agricultural Statistics Research Institute, Pusa Campus, New Delhi 110012, India

## Abstract

Halophilic archaea/bacteria adapt to different salt concentration, namely extreme, moderate and low. These type of adaptations may occur as a result of modification of protein structure and other changes in different cell organelles. Thus proteins may play an important role in the adaptation of halophilic archaea/bacteria to saline conditions. The Halophile protein database (HProtDB) is a systematic attempt to document the biochemical and biophysical properties of proteins from halophilic archaea/bacteria which may be involved in adaptation of these organisms to saline conditions. In this database, various physicochemical properties such as molecular weight, theoretical pI, amino acid composition, atomic composition, estimated half-life, instability index, aliphatic index and grand average of hydropathicity (Gravy) have been listed. These physicochemical properties play an important role in identifying the protein structure, bonding pattern and function of the specific proteins. This database is comprehensive, manually curated, non-redundant catalogue of proteins. The database currently contains 59 897 proteins properties extracted from 21 different strains of halophilic archaea/bacteria. The database can be accessed through link.

**Database URL**: http://webapp.cabgrid.res.in/protein/

## Introduction

The halophilic archaea/bacteria live in a variety of saline habitats. Halophilic microorganisms are traditionally defined as organisms that optimally grow in NaCl concentrations of above 0.2 M. Some of these halophilic microorganisms grow in NaCl concentrations of above 5 M. Halophilic organisms mostly fall in three classes with reference to salinity level optimal for their growth: halotolerant (1–6%), moderate (6–15%) and extreme (15–30%). Aerobic halophilic archaea have been extensively studied with reference to their physiology, ecology, biochemistry and bioinformatics.

Proteins can exist in globular or fibrous form depending on their function. A polypeptide is a single linear polymer chain of amino acids which are bonded together by peptide bonds between the adjacent amino acids. Halophilic proteins are known to be highly stable. These proteins are rich in acidic amino acids which are located predominantly at the protein surface. The three-dimensional structure analyses showed that most of the acidic residues are found on the surface of these proteins which facilitates excess protein hydration. This makes the surface more hydrophilic and more flexible. This in turn promotes nonspecific electrostatic interactions with salts in solution (1, 2). Acidic amino acids cluster on the surface of dihydrofolate reductase, proliferating cell nuclear antigen (PCNA) from *Haloferax volcanii* (3, 4) and glucose dehydrogense from *H. mediterranii*. Interactions between acidic residues on surface and hydrated salt ions not only prevent protein aggregation (5) but also maintain the functionality of the protein. Another strategy which increases hydration on the surface of proteins is making these surfaces deficient in lysine residues (6–8). Electrostatic stabilization is the key factor of halophilic adaptation of proteins. Ion pair or salt bridge is an important determinant of stability of proteins (9, 10). This is more so in the case of proteins adapted to extreme environmental conditions such as high salt or temperature. Interaction energy of salt-bridge could impart stability (11–13) or be destabilising for the protein (14–16) as shown by both theoretical and experimental studies. Mostly, halophilic enzymes function at 1–4 M salt concentration. This range is required for the stability and activity of halophilic enzymes (17).

Every protein has specific physicochemical properties. The deleterious effects of monovalent salts at multi molar concentrations on biological macromolecules from various organisms have long been noted (11, 18) and seems to be caused to a large extent by dissociation of groups, subunits, etc., which are involved in ionic linkages. If such ionic bonds are lacking in halophilic cell constituents, the physical chemistry of these structures must be unusual. In general halophilic structures were indeed found to be stable only in the presence of at least 1 M salt. In addition, most systems required or were stimulated by salt at concentrations near or even above this value. Thus, rather than being destroyed at high salt concentration, the macromolecular structures responsible for biological activity in halophiles appear, in fact, to be dependent on the presence of salts. A dramatic example of this unique salt dependence is the behaviour of the cell envelope of the halobacteria, when the salt concentration is lowered. Under these conditions, cells (12, 13) and isolated cell envelopes (14–16, 18–21) disintegrate to give slowly sedimenting fragments, and several membrane-bound enzymes are inactivated (22, 23). It is clear that, upon lowering the salt concentration considerable changes take place in the structure of the cell envelope and its constituents (24). The knowledge available in our database can be compared with non-halophilic archaea/bacteria and conclusions about fundamental mechanisms of survival in halophilic archaea/bacteria can be drawn in light of the above studies.

The current database contains 21 halophilic archaeal/bacterial strains. This database consist information about 59 897 proteins as listed in Table 1. Information about the physical and chemical properties of halophilic archaeal/bacterial proteins, such as theoretical PI, molecular weight, negative and positive charge, half-life of protein and amino acid index have been populated.

**Table 1:** List of different strains and number of proteins

S. NO	Strain name	Total number of protein information
1	*Azotobacter vinelandii*	10 414
2	*Bacillus cereus ATCC 10987*	10 691
3	*Halobacterium salinarium*	16
4	*Haloferax mediterranei ATCC 33500*	02
5	*Natronomonas pharaonis DSM 2160*	447
6	*Cellulosimicrobium cellulans*	08
7	*Haloferax volcanii*	462
8	*Haloarcula vallismortis ATCC 29715*	510
9	*Chromohalobacter salexigens DSM 3043*	6359
10	*Haloferax denitrificans ATCC 35960*	490
11	*Halorubrum saccharovorum DSM 1137*	6009
12	*Halorubrum distributum JCM 10118*	467
13	*Bacillus cereus G9241*	2480
14	*Salinibacter ruber DSM 13855*	5287
15	*Bacillus cereus E33L*	9914
16	*Chromohalobacter sp. HS2*	16
17	*Halorubrum lacusprofundi ATCC 49239*	459
18	*Halorubrum trapanicum*	01
19	*Salinibacter ruber M8*	5735
20	*Halomonas elongata DSM 2581*	127
21	*Chromohalobacter beijerinckii*	03
	Total	59 897

## Materials and Methods

### Source of data

**Figure 1.**
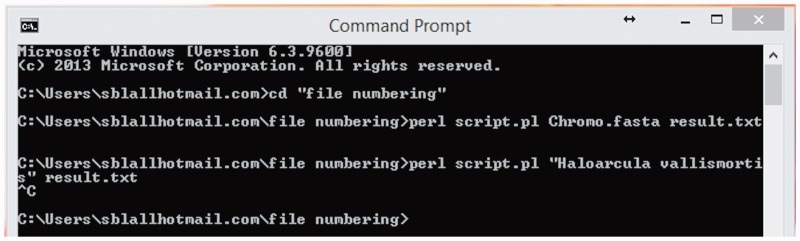
Snapshot of script run in Bioperl.

The protein sequences of different halophilic strains were downloaded from NCBI website http://www.ncbi.nlm.nih. gov/protein/?term=Halophiles+archaea. The Bioperl script was run for all halophilic strains. All biochemical composition such as Number of Amino acids, Instability index, Half-life, Number of atoms, Gravy, Aliphatic index were extracted through Bioperl script as shown in Figure 1.

pI or isoelectric point is the pH at which the net charge on the protein is zero. pI can be directly affected by the reduction of disulphide bonds in the proteins. The molecular weight is the elementary biophysical parameter and has direct correlation with the volume of the molecule. It influences the protein structure, which is functionally very important. The difference between the total number of positively (Arg + Lys) and negatively (Asp + Glu) charged amino acids in the protein gives the net charge of a protein. The pattern of hydrophobicity and net charge on the protein represents a unique structural feature of the proteins (20).

The half-life of a protein is defined as the time required for half of the total amount of protein in a cell to disappear after its synthesis. The *in vivo* stability of the protein is largely determined by the amino acids present at N-terminal of the protein and is given by the N-end rule (25–27). The instability index is an indicator of stability of a protein *in vitro*. The proteins with instability index smaller than 40 are predicted as stable, whereas, a value above 40 indicates instability of the protein (28) (http://web.expasy.org/protparam/protparam-doc.html). The formula of instability index (II) is as follows:

    i=L-1
II = (10/L) * Sum DIWV(x[i]x[i+1])
i=1
where: L is the length of sequence
DIWV(x[i]x[i+1]) is the instability weight value for the dipeptide starting in position i.

The relative volume occupied by aliphatic side chains (alanine, valine, isoleucine and leucine) is defined as the aliphatic index of a protein. The aliphatic index may influence thermostabiltiy of globular proteins. The sum of hydropathy values of all the amino acids, divided by the number of residues in the protein sequence gives the GRAVY value.

### Database architecture

**Figure 2.**
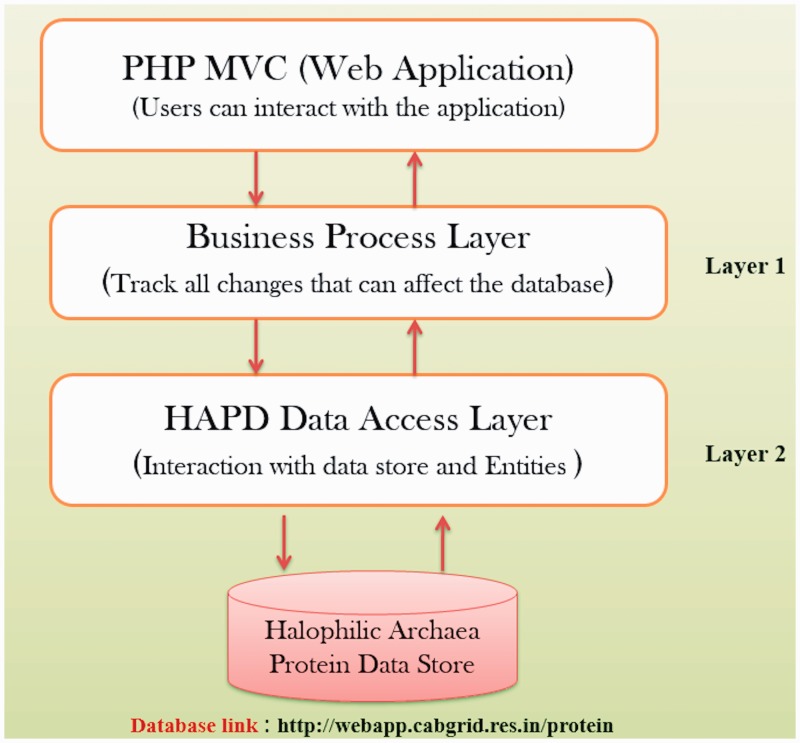
HProtDB architecture.

In order to store the information about protein properties of different strains of halophilic archaea/bacteria, open source database software MySQL (version 5.1.3.6) was utilized. The data is stored in the form of associated tables, which also follows Relational Database Management System (RDBMS) concepts. MySQL is feature-rich database software that provides speedy data access, ease of use, portability and also supports most of ANSI SQL commands. The data consistency and non-redundancy were maintained by employing normalization techniques on the developed database. HTML and PHP were used to render a dynamic web interface and the appropriate database connectivity techniques were utilized for quick and easy information retrieval. The viewing of the data is freely available along with a facility to download data. This web application has been hosted using an open source WAMP Server (version 2.0i, windows web development environment) which also provides multiuser access facility. WAMP server allows hosting web applications developed using PHP and MySQL over Apache2 web server. Figure 2 depicts the architecture of HProtDB.

The spectrum of the database comprises of database tables for user management, protein, biochemical and biophysical properties of proteins. Besides, fields of the tables cover details of all attributes of the concerned parameter. A primary key in each table is identified for uniquely defining a record. Similarly, the foreign keys were identified from other tables for setting relationship among different entities. Some of the tables were master tables, which were meant for providing the real world values to fields in different tables, while building the queries and presenting the reports.

**Figure 3.**
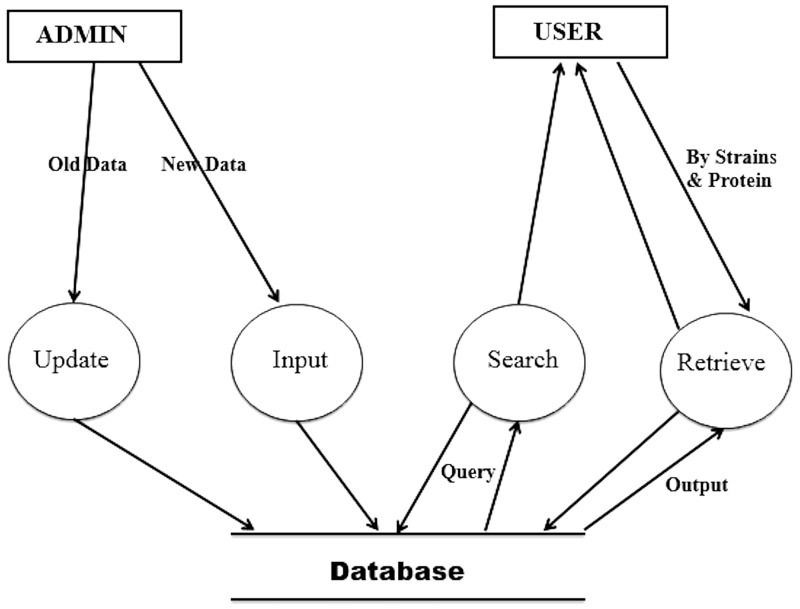
Data flow diagram.

Figure 3 shows the Data Flow Diagram (DFD) of the HProtDB. The whole system has been depicted in such a way so that the continuity of information flow should not be lost at the next level. This DFD shows all the processes together with the data stores.

**Figure 4.**
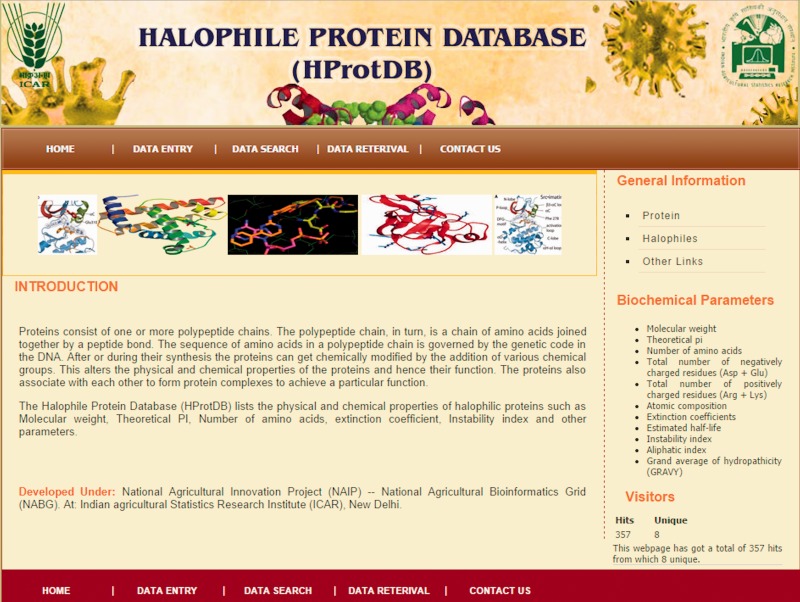
Screenshot of the Halophile Protein Database (HProtDB) home page.

**Figure 5.**
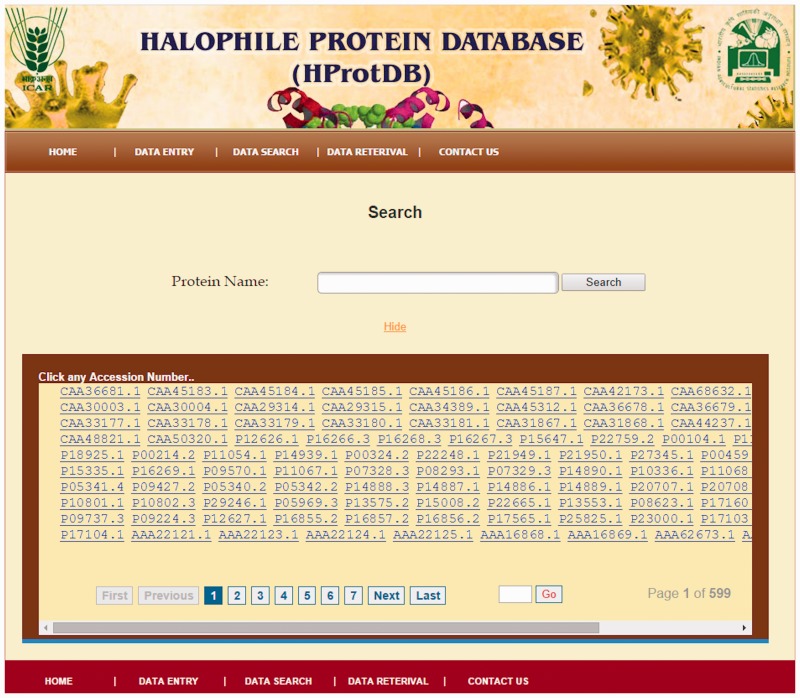
Search page of HProtDB.

**Figure 6.**
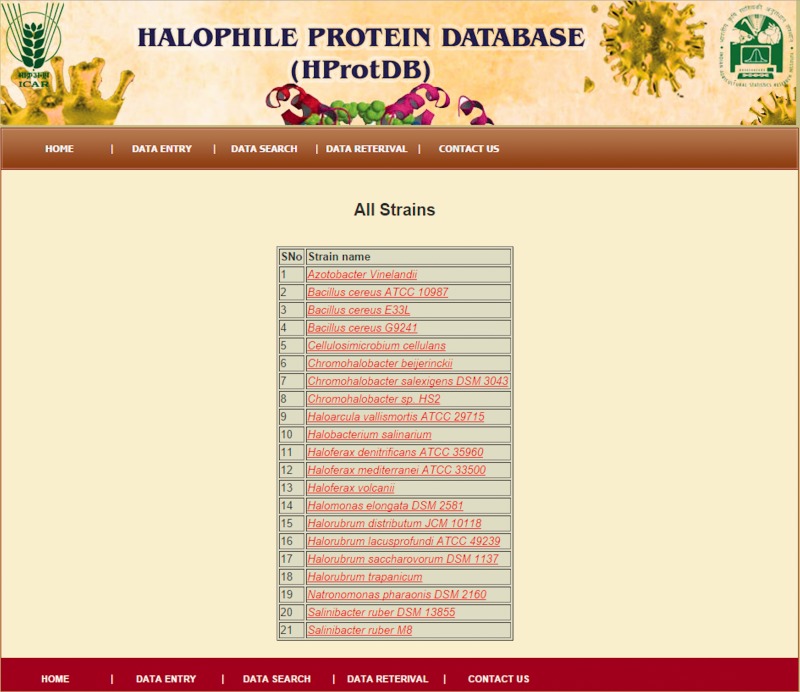
Snapshot of different strains list.

**Figure 7.**
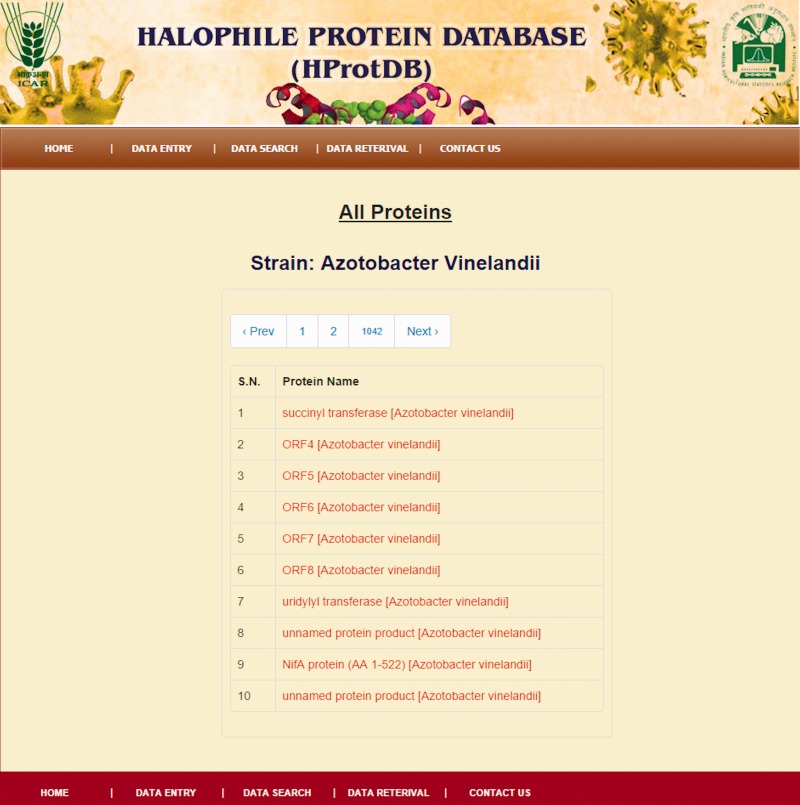
Snapshot of protein names of specific strains.

**Figure 8.**
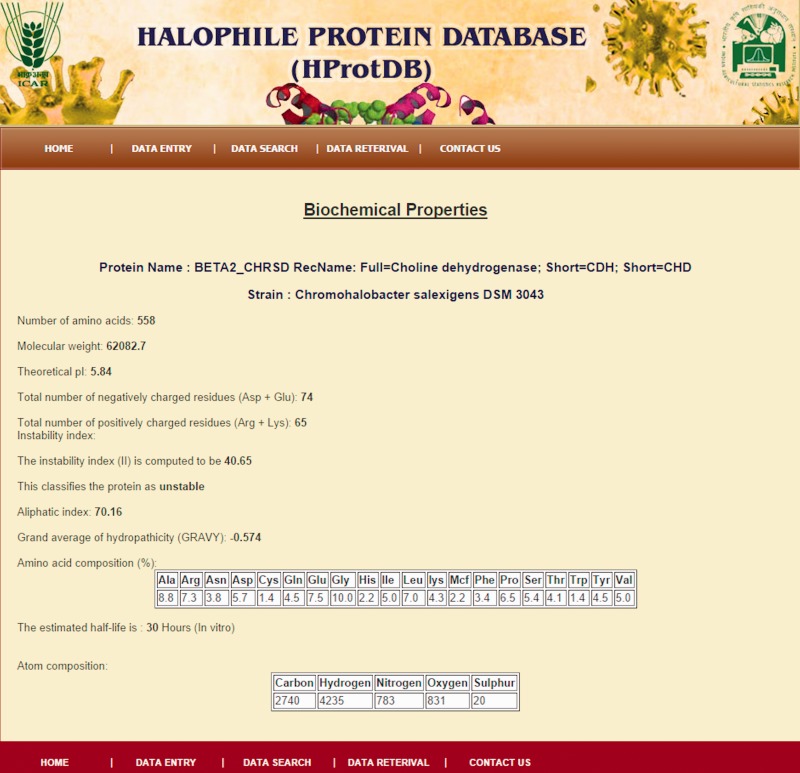
Snapshot of biochemical/biophysical properties of protein.

The home page of the database is depicted in Figure 4. The different tables on the home page provide links to general information, such as protein, amino acids, microbes and other modules related to data entry and retrieval. The search facility (Figure 5) enables the user to search the biochemical and physical properties of the desired protein either through accession number or protein names given in the dropdown list. The user has to select the desired protein, and subsequently all information related to the protein gets extracted from the database and displayed on the screen. The data retrieval option on the home page also provides the user to search for any specific halophilic archaea/bacteria records. This option provides the list of strains and clicking on a particular strain gives the protein and protein properties. In this way, user can access any or all 21 different strains of halophilic archaea/bacteria (Figures 6–8).

## Results and discussion

We have constructed a database which provides biochemical/biophysical properties of the proteins from halophilic archaea/bacteria. The study of these properties may lead to elucidation of mechanisms for salt tolerance. Identifying salt-tolerant proteins in halophilic bacteria and transfer of such proteins to other agriculturally important bacteria such as *Rhizobium, Azotobacter, Cyanobacteria etc.* will be useful from applied point of view as the engineered microbes may be able to adapt in saline conditions. The information in our database may also be useful for designing synthetic proteins with optimal physicochemical proteins which may be of use in saline conditions.

## Conclusion

The HProtDB lists various physicochemical properties of the proteins of halophilic archaea/bacteria. Halophilic archaea/bacteria are excellent models for study of osmoregulatory mechanisms that permit these organisms to grow in saline environments. The information in the database might prove useful in elucidating the fundamental mechanisms for salt tolerance and for identifying the characteristics of the genes involved in salt tolerance. These may prove useful in identifying and annotating novel salt tolerant genes (29).
